# “The state They're in”: Unpicking *fantasy paradigms* of health improvement interventions as tools for addressing health inequalities

**DOI:** 10.1016/j.socscimed.2020.113047

**Published:** 2020-07

**Authors:** Mhairi Mackenzie, Kathryn Skivington, Gillian Fergie

**Affiliations:** a Urban Studies, School of Social & Political Sciences, University of Glasgow, 27 Bute Gardens, Glasgow, G12 8RS, Scotland, UK; b MRC/CSO Social and Public Health Sciences Unit, University of Glasgow, UK

**Keywords:** UK, Social determinants of health, Health inequalities, Social prescribing, Structural competency, Primary care, Qualitative research

## Abstract

Globally, it is recognised that the fundamental causes of iniquitous health outcomes lie within unequal distributions of wealth and power. Internationally, however, policies and interventions persist in individualising the inequalities problem and targeting individual behaviours as the main solution. This approach has been argued to represent *‘Fantasy Paradigms’*. This paper explores one example of such *‘Fantasy’* intervention from the perspective of health practitioners. Further, it explores opportunities for deepening practitioner understandings of the socio-political determination of health. Data were collected through in-depth interviews with 47 professionals involved in delivering a social prescribing programme in poor areas of Glasgow, Scotland. Data were analysed thematically across and within transcripts. Narratives highlighted different explanatory types concerning how the intervention could tackle health inequalities including: firm commitment to individualised approaches; *hopeful pessimism;* the *social-determinants-of-health* as an unpoliticised and nondeterministic backdrop to poor health; and finally, incomplete understanding of the social gradient as a population concept. Disrupted narratives of the social determination of health were also evident. This paper contributes new insights to existing debates on health inequalities discourse. These are conceptually important and identify opportunities for sharpening practitioner understanding of the social determinants of health which could in turn contribute to better, non-stigmatising primary care. It argues that re-engaging communities of practice with what is meant by *determination of health* is necessary and that there is a need to *de-couple* the policy aim of reducing health inequalities from the delivery of structurally competent and equality-focused public services.

## Highlights

• Why are individually-focused interventions thought to reduce health inequalities?
• From our data, we identify believers, ‘hopeful pessimists’, and dissonant discourses.
• Lifestyle-oriented understandings of health problems and their solutions prevail.
• Important opportunities for deliberative dialogue and agenda-setting are identified.
• Decoupling health inequalities policy from health mitigation of poverty is advocated.

## 1 Introduction

There have been calls to reframe thinking about the social determinants of health inequalities due to the gap between evidence and policy; the former focused on socio-economic circumstances, the latter on lifestyle behaviours^1^ (Elwell-Sutton et al., 2019; Phelan and Link, 2015). Scott-Samuel and Smith, indeed, argue that policymakers who devise sub-macro level interventions to reduce health inequalities (HIs) are guilty of pursuing ‘*fantasy paradigms’* (2015). This is a devastating critique for policymakers with commitment to social justice. The argument, however, is two-fold. First, blinkered approaches to the evidence base concerning HIs causation lead to policy-making repertoires focused on responsibilisation and lifestyle change which merely nod to inequalities' socio-political backdrop. Second, even where this backdrop is acknowledged, the pervasive, health-damaging political context (in the form of decades-long neo-liberal policies and practices) ensures that more progressive policies swim against the tide. Scott-Samuel and Smith argue too that researchers collude in fantasy production through engaging in evaluation of such policies.

Although as evaluators we are implicated in this critique, we are nonetheless sympathetic to the arguments. Here, we develop these empirically and conceptually by exploring ‘*fantasy paradigms*’ using the case of a social prescribing intervention, the Links Worker Programme (LWP). Using data generated in the evaluation of this intervention (based in Scottish General Practices covering populations living in the areas of most concentrated deprivation), we set out different types of thinking behind practitioner commitment to such interventions *as a means of reducing inequalities*.

To be clear: this paper's purpose is not to downplay the significant role that publicly funded primary care in the UK has played in addressing HIs since the introduction of the NHS in 1948. A publicly funded primary care system, well-resourced according to need and with committed practitioners, is paramount to narrowing the gap (Watt, 2012). Nor is it our intention to comment critically on social prescribing programmes *per se* - often designed with deep recognition of socio-political determinants in mind (Cawston, 2011)*.* Our interest is the role that interventions such as these are perceived to play *in the reduction of HIs* since, as Watt reminds us, if ‘delivered inequitably, health care can widen inequality’(2012:p.3). We use *‘fantasy’* to indicate how interventions such as the LWP are considered by practitioners to mitigate or tackle HIs; in other words, to understand practitioners' support for the kind of intervention described as a ‘*fantasy paradigm’*.

Through a process of abductive analysis (Tavory and Timmermans, 2014), we categorised these emergent explanations as: belief that poor health is down to individuals and best addressed through individualised solutions; and a view of such interventions as part of a hazy, bigger, and sometimes unarticulated, solution to HIs. This second category consists of: *hopeful pessimists* - those holding out against the odds for *mitigation potential;* those for whom the *social-determinants-of-health* operate as a strangely unpoliticised and nondeterministic backdrop to poor health; and finally, those appearing to misunderstand the social gradient as a population concept. As identified in previous work (Babbel et al., 2017; Mackenzie et al., 2017b), we also highlight disrupted narratives of the social determinants of health (SDoH). These include inconsistencies in what individual participants say, between participants within focus groups and, relating to particular policies. This latter point concerns the way in which consideration within interviews of recent Conservative-led welfare ‘reforms’ in the UK, produced more discussion of the material drivers of ill health than did other policies.

This paper, the first to explore the concept of ‘fantasy paradigms’ empirically, starts by outlining the *‘Fantasy Paradigm’* position with its backdrop of nearly 50 years of HI research. We then set out the LWP case, before summarising our methods and presenting our analyses. We conclude by reflecting on the implications for Scott-Samuel and Smith's thesis concerning a realistic agenda for future academic, policy and practice HI work. This includes the need to decouple narratives of *mitigating the effects of the SDoH* and *tackling the fundamental causes of HIs*. This latter point is important in ensuring that public services are properly supported and legitimated as a means of strengthening safety nets for individuals especially when they are under sustained political threat (Taylor-Gooby, 2013).

### 1.1 The fantasy paradigm thesis in a nutshell

We do not rehearse the literature on HIs as a phenomenon (Graham, 2000; Black et al., 1980; Marmot et al., 2020), as a policy failure (McCartney et al., 2013), or as contested discourses (Raphael, 2011; Smith, 2013a). It is long recognised that the fundamental causes of HIs rest with differentials in power and capitals of various sorts invested in socio-economic status (Phelan et al., 2010), and that there exists a lack of evidence for the success of individualist interventions in tackling HIs. This operates as a backdrop to the Fantasy Paradigm argument. Previous commentators have pointed to failure being due to public health interventions designed at the wrong level (Blackman et al., 2010; Lynch, 2017; Graham, 2009) even when government rhetoric itself points to the need for macro-level action (Smith and Hellowell, 2012)and to ‘lifestyle drift’ (Katikireddi et al., 2013). The latter denotes the idea that even interventions with structural sensibilities tend to shift, in their application, to ‘easy-win’ action at the individual level.

Scott-Samuel and Smith develop this further. They argue that, in conditions of advanced neo-liberalism, policy interventions as currently conceived cannot tackle HIs – *whilst policies may be desirable in other respects*, they are destined to fail in reducing the gradient in health, due to counter-forces. Further, they argue, instead of viewing those who advocate for reimagined futures (where inroads into disrupting the structural determinants of health are possible) as fantastical, charges of fantasy should be levelled at policies and strategies which claim potential to achieve reductions in HIs via lifestyle or health-service mechanisms. Thus, they argue, the development and evaluation of individually-focused interventions represents collusion (witting or unwitting) in sustaining *Fantasy Paradigms*. The fantasy paradigm operates through the continued belief that population-level inequalities created through larger global/national structures can be eradicated at local/individual levels. Finally, they suggest that, rather than view utopia as a pejorative term denoting disconnection from the real world, researchers should adopt utopia as method, as per Levitas (2013), as aspirational spaces working through policy, practice and community networks to open up reimagined futures. Such endeavours involve: utopia as ‘archaeology’ – scholarly digging into what policies and discourses reveal about the framing of policy problems; utopia as ‘ontology’ – where scholars, working with other citizens, endeavour to understand the world as it is and consider how existing economic and social structures differentially enhance the opportunities and capabilities of communities; and, utopia as ‘architecture’ where collaborative approaches envisage alternative futures and the steps conducive to their construction. Utopias, in this sense, are not fixed and ideologically singular but plural and contingent.

Scott-Samuel and Smith's paper explicitly focuses on national government policy in relation to HIs; other work by Smith and colleagues investigates reasons for policymakers and researchers to support individually focussed interventions (Garthwaite et al., 2016; Smith, 2013b; Smith and Kandlik Eltanani, 2015). In these analyses, real politik and a belief in insufficient public appetite for reducing inequalities contribute to the continuation of such interventions and their evaluation. This paper drives the argument forward by examining how policy interventions —in this case, a social prescribing intervention—are understood by its key practitioners to address poor health and tackle HIs (archaeology in Levitas' terms). It asks, for the first time, what are the components of the *fantasy* paradigm that exist on the ground and what do they tell us about opportunities for moving to deeper practitioner understandings of the actual socio-political determination of health?

### 1.2 Social prescribing and the Links Worker Programme

Faced with stubborn inequalities and recognising that many health presentations to primary care have underlying social causes, social prescribing is generating considerable interest within the UK (Cawston, 2011). In social prescribing (analogous to pharmaceutical prescribing), GPs and other primary care team members, make diagnoses that pay heed to social determinants (such as loneliness or financial difficulty), and develop ‘social prescriptions’ to address these. This involves linking to services better suited to support such needs. Referrals might be made to, e.g., third sector organisations offering smoking cessation, money advice, befriending, or exercise classes.

Alongside these developments, however, the organisations that deliver many of the proposed ‘prescriptions’ (e.g. libraries or community centres) are experiencing funding cuts (Finch et al., 2018). In the context of austerity-driven public and third sector funding cuts, the proposed model of social prescribing is, argued, therefore, to be flawed (Skivington et al., 2018; Wildman et al., 2019).

The evidence for social prescribing efficacy is limited because of the lack of good quality studies (Bickerdike et al., 2017) and because the programmes suffer from considerable implementation challenges (Bertotti et al., 2017). The cluster-randomised evaluation of the LWP showed no effect on its primary outcome (health-related quality of life) or on secondary outcomes related to mental health and health-care utilisation although per protocol analysis showed reduced anxiety for those with more than two CLP visits (Mercer et al., 2019). Whether the evaluation was long enough to capture change is open to question; Watt argues that sustained investment is required for the public health benefits of primary care investment to accrue (2012).

Nonetheless, recommendations for programmes persist, and policymakers pledge support to extend these types of services in England (NHS England, 2019; Royal College of General Practitioners, 2018) as a tool in redu*cing HIs*.

The LWP initially aimed to tackle HIs but, very quickly after its evaluation was commissioned, policymakers realigned objectives relating to HIs to improving health outcomes (Health and Social Care Alliance Scotland, 2013; NHS Health Scotland, 2016). Briefly, the LWP employed Community Links Practitioners (CLPs), as an additional resource with community development expertise based in GP practices: to engage with ‘vulnerable’ patients and to connect them with community resources; to act as a channel between GP practice and community services to support organisational links; and to foster change within General Practice. They would thus countermand the additional pressures of workload and complexity of cases found to prevail in practices in poorer areas (O'Brien et al., 2014; Popay et al., 2007). Ultimately, however, intensive work with individual patients took precedence over the components concerning community and practice development, with the latter focussing largely on staff well-being (Mercer et al., 2017). The focus shifted from ‘linking’ patients to community resources to ‘fixing’ patients (Mercer et al., 2019).

The ways that professionals tasked with implementing the LWP discuss HIs and the role of social determinants in creating poor health, offer a window onto the *Fantasy Paradigm* hypothesis from a practitioner perspective.

## 2 Methods

The findings are drawn from data collected in the process evaluation of the LWP (ethics approval granted by the University of Glasgow, College of Social Sciences Research Ethics Committee) GPs and CLPs were interviewed individually and in groups with a larger group of professionally qualified primary care staff including, for example, practice managers, practice nurses, and representatives from community organisations (total N = 47). In total we analysed 12 individual interviews and 15 group interviews. Group interviews varied in size from 2 to 6 participants.

Interviews were conducted with participants from fifteen GP practices within Glasgow, a post-industrial city with the poorest health in Western Europe (Baruffati et al., 2019). Seven GP practices had been randomly allocated to the LWP and eight formed the comparison group – collectively they served some of the most socio-economically deprived populations in Scotland. Both intervention and comparison GP practices had volunteered for the LWP, and were, therefore, open to delivering this type of social prescribing programme and knowledgeable about its purpose. As we discuss later, these practices are members of so-called Deep-End practices referring to the concentration of poverty and other markers of deprivation within their practice areas and to the increased challenges of serving populations in these practices. Deep-End serves as a descriptor of practice demographics but is also important to this study because some of our participants may have been engaged in advocacy work on behalf of GPs (e.g., lobbying for greater resources); some may also have been engaged in advocating directly on behalf of patients (for example, calling for welfare changes). This positions our participants as those most likely to be informed about the SDoH and of the importance of non-clinical health protective resources (as per Phelan et al., 2010).

Participants were interviewed about their experiences and views of the LWP. Interview topics included the role of the CLP, the scope of the LWP in supporting patients and its relation to HIs and, the potential of the LWP to create change both within the GP practice and the community. It would have been useful to gather data on professionals’ own socio-demographic backgrounds; unfortunately, this was not feasible within the range of data collection required for the evaluation. Further information on recruitment and sampling, including ethics and informed consent, is reported elsewhere (Mercer et al., 2017).

The authors jointly reviewed a sample of the transcripts; an initial coding framework was agreed through discussion of emergent themes. The coding framework was applied to all transcripts by one author and checked by another. Transcripts were coded and analysed manually because of the manageability of a relatively small dataset (Basit, 2003). Themes were developed abductively through discussion in regular data-surgeries and review of the coded transcripts (Tavory and Timmermans, 2014). Situated between inductive and deductive analyses, abduction moves between data and prior conceptual frameworks to test and expand analytic categories. From the HIs literature we were attuned to two types of explanations of how individually-focused change might tackle poor health and HIs. These were: primacy of individualised problems and solutions to poor health; and a view of such interventions as part of a bigger solution. The subcategorization of this latter narrative was generated abductively as analysis progressed. In addition, it emerged that there were different forms of disrupted narratives evident in the data. This took the form of inconsistency in individual narratives, between participants of group interviews (we were alert to the ways in which group narratives were constructed through in-group differences) and relating to specific policies. Comprehensive coding notes were written for all transcripts, using headings derived from these themes. All coding notes were checked for agreement against the transcripts.

## 3 Findings

Findings are organised according to key themes emerging from our analysis. First, that poor health and its remedies rest with individuals, though in some cases recognising the need for practitioner support. Second, perspectives that acknowledge some role for socio-political factors in creating health and inequalities and that square this (sometimes inconsistently) with the contribution of the LWP. Third, we present findings about disrupted narratives of the SDoH. As above, we use the term *fantasies* in relation to Scott-Samuel and Smith's argument about fantasy paradigms. *This does not imply that interventions responding to the health needs of individuals living in poor areas are not fundamentally important but that their connection to reducing inequalities is problematic.*

Verbatim quotes are attributed to professionals (GP, CLP, Practice Nurse, and Community Organisation Representative) within the uniquely identified comparison (C) or intervention (I) practice i.e. GP from comparison practice 1: GP:C1. Our findings did not differ by programme receipt nor by practitioner type.

### 3.1 Dedication to individualised problems

This section sets out the dominant discourse within our data: poor health is caused by poor lifestyles and/or negative dispositions. It represents the ultimate *fantasy* paradigm in Scott-Samuel and Smith's terms since it is profoundly disconnected from evidence about the socio-political context of health. We highlight aspects of this *fantasy* derived from our data: health is created through individual choice; the target population for social prescribing interventions is ‘other’ compared to professionals and the wider population; and, where the broader causal frame for understanding HIs is surfaced, it does not connect individuals with their socio-political context.

First, participants in individual and group interviews talked of behaviours in which people engage. As one GP says, the “fundamental problem is to do with diet, education and exercise” (GP:C3). The use of ‘fundamental’ is especially interesting given the literature which sets out the fundamental causes as the unequal distribution of power and wealth (Link and Phelan, 1995). Changing health behaviours was viewed as a matter of choice, though with the intertwined argument that the target population are deficient in confidence and ability to form appropriate social networks (thus requiring support to effect individual-level change). The lack of will to change was viewed as health professionals' ultimate challenge. Relatively rarely in our data was it connected to meaningful recognition of wider social circumstances, or to income and deprivation. Instead, the frequently expressed view was that the CLP should demonstrate where responsibility for poor health lies:

“… making them absolutely question themselves -why their health is in such a way. And who was responsible for that and how can they actually make things a wee bit better for themselves?” (CLP:I5)

The nub of the individual choice *fantasy* is that, with support in navigating systems, individuals can deal with issues affecting their health. Those choosing not to make changes are thought lacking in agency and need to be supported to “learn how to take responsibility for looking after themselves” (PN:C3).

The role of social prescribing was elaborated as being “[to] build their confidence up and get them planning for the future” (CLP:I2). Practical examples of this were provided throughout the interviews: walking groups, community cafes, community allotments, and cookery groups. Patently, the existence of these resources is important in sustaining individual and community health (within fundamental causation theory, they might be viewed as health protecting resources), but the point here is that they were not understood *to be part of a wider economy of health production*.

The second thread to this *fantasy* narrative is that the target population is different from the rest of the healthy population. This was conveyed tacitly by an invocation by some participants to the interviewer to acknowledge a category of ‘these people’ (a facet likely exacerbated by largely middle-class professionals working, but not living, in poorer areas).

“the reality of the world is these people won't do it [access resources] by themselves, it's a function of the state that they're in that they won't be able to do it for themselves, they won't think of doing it for themselves” (GP:C3).

It is ironic that ‘the state that they're in’ is used to signify individual deficits rather than to describe the socio-political context for poor health. The target population: “doesn't have the ability to figure things out for themselves” (GP:I5). Further, they need to work with a professional who can bridge a communication divide—thus, the CLPs “need to know how to communicate with these people … it's about speaking a different language, or a … dialect or patois” (GP:C3). Further this GP alluded to the view that the CLPs themselves form a class bridge, thus “they can't be middle-class”.

The most extreme example of the tendency to ‘other’ the target population is provided by a GP who discusses the difficulty in enacting health improvement approaches:

*‘they're difficult in terms of the way they interact as a personality. Which generally means that they're coming from a more complex, deprived background. Because we're now getting back to the people who are working with their limbic pathways and not using frontal lobe. And you can't have rational discussions with them.”* (GP:I6)

Of course, not all participants talked in these terms. There was also evidence of empathy with patients:

“If you actually look back and let the person talk and be open about it … the person will have experienced … trauma or … something … that will have a massive impact in their resilience and the way that they're able to self-manage … the complex issues that people face cannot be solved overnight” (CLP:I6)

And, as discussed in the next section, there are narratives presented which fully acknowledge the narrow scope of health improvement in improving the lives of those at the bottom of the gradient.

Later in the findings section we expand on how narratives of poor health and inequalities are inconsistent, but here we draw attention to the third signifier of this *fantasy* narrative. This is that even where the SDoH were mentioned, they were sometimes curiously divorced from how individuals' health trajectories are *determined by the social.* This is illustrated by one focus group discussing how to encourage uptake of various preventive health programmes: “there's lots of empty shops across the road, and having … you know, like having advertising for cervical screening, for breast screening, for bowel screening, for heart disease checks*”* (PN:C3). Empty shop fronts are here presented as an opportunity for advertising screening rather than symbolic of wider social issues *fundamentally related to poor health.* Likewise, whilst decent housing and food were discussed as being prerequisites to good health, the solutions posited to their absence were concerned with providing people with the ‘confidence’ to remedy these deficits in their lives. Thinking socio-politically is not at the forefront of thinking; a phenomenon likely related to GPs experiencing health problems through individual presentations.

Taken together, the message of this *fantasy* narrative is clear. Health is an individual responsibility and its improvement (including in relation to disparities between individuals) is a matter of choice albeit one that parts of the population need help to realise. The social and political determinants of health are mentioned as though they are somewhat free-floating phenomena rather than actual determinants.

### 3.2 Intervention as part of a bigger, hazy solution

The second *fantasy* narrative that we highlight (and in which we are partially implicated as evaluators), is a set of arguments about how interventions such as the LWP might mitigate the effects of the SDoH at the level of the individual or community. Again, we remind the reader that we use *fantasy* to denote a category of discourse that may describe good primary care practice but where connections to reducing HIs are more tenuous. In this study, these arguments are not a cogent set of arguments about causal pathways and, indeed, include identifiable errors of logic. What they have in common is some concern with inequalities as an issue that should be attended to. Here, we discuss the following expressed ideas: there is a need to do something even if there is evidence to suggest it may not work (hopeful pessimism); it is expedient for services to capture funding in times of austerity; changes at an individual level can equate to wider change through a ‘ripple effect’; and, relatedly, the gradient can be altered one person at a time. We also identify a lacuna in the data; as *hopeful pessimist* evaluators we expected to see narratives of the intervention operating through increasing practitioner awareness of the SDoH as played out in the lives of individual patients, thus offering opportunities for more structurally engaged encounters with patients. This potential mechanism for mitigating the negative impacts of the social determinants connects to Freese and Lutfey's (2011) idea that institutional practices have attenuating or exacerbating influences regarding the fundamental causation of HIs. Of this type of narrative, however, we saw relatively little.

First, the hopeful pessimist narratives recognised the fundamental causes of poor health. Thus: “You can't deal with health and social issues without talking about politics because that's what's defining it […] the social determinants won't shift because the social determinants are going to stay with a UK government hell-bent on austerity” (GP:C8). This respondent also demonstrated awareness of how such social determinants shape the parameters within which choice might be deployed:

“life's not great, they've got very little in the way of money now and they're being squeezed and sanctioned ‘til they're blue in the face. They haven't got a job, they probably have to go and get a job, if they do … it'll be like a zero-hour contract below the living wage … and so, to try and do that little bit extra about trying to live a more healthy lifestyle can just seem a bit pointless. Why bother?”

Using the familiar metaphor that “we're trying to … pull people out the water downstream”, this GP believes that intervening, as per the LWP, might operate at the individual level (through encouraging positivity that might lead to a healthier lifestyle, improved medication and appointment uptake through to community involvement). S/he concludes “maybe it’s a bit idealistic but … it feels right to me”. It is also fundamentally disempowering for professionals themselves to practice with a *‘why bother’* mindset and so action at the individual level is deemed better than none at the political level - “you'll wait a long time for political solutions” (GP:I1). As this GP continues:

“I'm quite involved in politics and that's partly because people sit in front of me and I can almost very rarely see a medical solution to their problems. It's almost always about money, benefits, inequalities, social barriers, sectarian barriers … stuff that actually is more of a political or a public health solution than there is a GP solution. So that's how I keep sane cos I think, I can't help people that effectively as a GP. I can do bits of things [our emphasis]”.

A second reason for GPs engaging with such interventions is that they bring additional sources of funding that may be viewed as the only way to secure partial solutions in crisis situations. This is important when GPs are dealing with individuals with harrowing difficulties exacerbated, in the context of long-term austerity, with swingeing cuts to social security safety nets, public and third sector services. In justifying this type of intervention, one CLP provided an example of the support provided for a man with a learning disability in crisis brought about by falling through social service and housing support nets. Through detailed casework and connecting with other services, the practitioner intervened to ensure that this man had better, more supported housing. Examples of this type, in the context of shrinking budgets more generally, can understandably lead to a desire for this type of funding to continue and be extended. For example, the GP who had argued that politics define health, said:

“I'm hopeful that … because there is a big interest in trying to do something about inequalities in health that you will continue getting funding for projects like this … I hope it does very well and that it’s rolled out bigger and better” (GP:C8).

Not all who foregrounded some aspects of the SDoH saw health service interventions such as the LWP as only being about mitigation of poor circumstances. Others also stated a belief that HIs would diminish as a result. One GP argued that: “The whole ethos of what the LWP is, is to ultimately address health inequalities” (GP:I2). Another expressed the view that it would “have a really sizeable impact” (GP:I5). An explanation of these views is perhaps that it is not in the interest of GPs under significant workload strain to argue against additional funding and, indeed, one explicitly states that “If you want to address health inequalities, you need to be pushing, putting resources into Deep End practices” (GP:I2).

Another explanation rests on misunderstanding the difference between mitigation of negative social effects at the individual level and levelling the health gradient at a population level. Such misunderstandings were evident within the data and are likely symptomatic of working with patients as individuals. The first manifestation of this was in the deployment of the ‘ripple’ effect as an explanation of how HIs could be tackled through individual-level intervention:

“Small changes in human behaviour and interaction make big changes further down the line, the butterfly flaps its wings and then you have a hurricane … in North America.“. *(GP:I6)*

The ripple effect argument explained how one positive change might lead to another for individuals and across generations and networks. Individuals with the help of CLPs become, in this scenario, responsible for their own health improvement and sow the seeds of wider change. Intra-individual ripples are captured as follows:

“they've got ten things that are stressing them, …getting them to put them in rank order and then starting at the wee-est one first and chipping away at the more chipable away-able stuff, can make a big difference because their overall burden diminishes […] might have had a big effect on how people felt they could then approach bigger things” (GP:C8)

Further, the ripple effect idea was considered to have a wider effect in the community. “[L]ike a lightning conductor” *(GP:C3)*, it could get more people within the community involved in local-area groups, ultimately leading to improved health and reduced inequalities:

“maybe they would all of a sudden change and they weren't smoking or they weren't drinking. But, you know, but they had that kind of confidence, people say, well, if they can do it then I can do it, and also maybe it would challenge kind of entrenched ideas the community might have of maybe being a bit rigid or not doing anything different or that there would be no value in breaking out of the mould or the roles that they've been given, that they could do it and maybe the Links Worker would be the catalyst” (GP:C4).

Much of this implied effect is expressed as aspiration rather than evidence and might be viewed as a health parallel of the ‘trickle down’ fallacy of economic growth, identified by Scott-Samuel and Smith (2015) as a component of the *fantasy paradigm* of current political thinking.

A further issue is how the inequalities gradient is conceptualised. The distribution of health outcomes by social class across a gradient is a relational concept yet it was articulated by one participant as a fixed, physical ladder that one might climb (presumably over the shoulders of others). The quotation also hints at the worldview noted by Scott-Samuel and Smith whereby turning the working class into the middle class is tacitly identified as part of the solution of inequality. Thus, s/he would:

“turn my patients into what middle-class patients would do if they had the same problems. I want to put them up three grades in the, five grades, whatever grade, in the socio-economics you know, group, that's what I want to do” (GP:C3)

If the concept of the gradient is confused, then it is no surprise that there is difficulty in recognising the difference between mitigating the negative effects of the social determinants and tackling the root causes of HIs; thus the two are conflated. From this comes a view that it “doesn't matter that you can't help a million people, if you help one … you're going to give a legacy” *(CLP:C3).*

Finally, an important strand of argument in this *fantasy* paradigm, is that a CLP working with individual patients, community groups, and the GP practice will bring structural competency to interactions with patients and to the task of advocating (even politically) on their behalf. At the very least, such structural competency displayed at the micro-level encounter between patient and health care professional would mean that health service encounters themselves would not further stigmatise and place undue weight on individual responsibility. As indicated earlier, it is this aspect of such interventions that we as evaluators believe offers some promise of actual mitigation for individual patients. However, as discussed, the LWP strands of community and practice development were less fully attended to than work with individual patients. Only one participant set out the political causes of poor health and connected this to a limited mitigation role of what the LWP could realistically achieve. When asked about HIs, this CLP laughs and states that the “fundamental issues around poverty and deprivation have been around for years and it's gonnae take more than a links worker …” (CLP: I2)*.* S/he then captures the role as mitigating the effects of the SDoH, particularly in the context of depleted public services, but also identifies the need to educate others:

I02) “Their training doesn't lend itself to, you know, understanding, necessarily. Maybe it’s a bit harsh but understanding of issues affecting, you know, poverty, drugs, addiction, you know, all these kind of things. So, sometimes they can be quite dismissive of patients or a bit short with them and then, so, there's opportunities for me to say “Well, actually, you know, that person's life hasnae been that straightforward”. So, there's scope for wee, kinda, bits like that. And you can see people, kinda, going “Oh. Oh.” You know, and kinda taking that on board. But to say that there's been significant changes? No.” (CLP:

While there were examples of participants attending to the impact of social and economic problems that flow from political decision-making, and of investing time in finding partially mitigating solutions (the debt and housing crisis example described above), there was also a common narrative that CLPs were additional resource to deal with the ‘social’. This implies that those GPs so inclined can be legitimised in retreating to a medical model of care assuming that ‘the social’ is dealt with elsewhere. This is understandable given the increasing workload pressures that GPs find themselves working under (Baird et al., 2016) - specifically, those working in poorer areas where work is described as an ‘endless struggle’ - (O'Brien et al., 2014). It is, however, at variance with structurally competent practice as advocated by Metzl and Hansen (2014), where family doctors are encouraged to understand health outcomes in structural terms and to act accordingly.

## 4 Disrupted narratives

Finally, we turn to a line of argument that is consistent with earlier work on HI narratives (Babbel et al., 2017; Mackenzie et al, 2017, 2017a). Narratives of how social, economic and political factors determine health are not only contested but are rarely consistent. Frame inconsistency tells us about tensions within paradigms and, correspondingly, potential spaces for deliberative debate. Some examples within individual narratives have already been highlighted, particularly relating to the likelihood of social prescribing plausibly tackling HIs. Here we illustrate tensions in the group discussions and highlight one interesting feature of our data – that UK government welfare ‘reform’ occasions mention of material and social drivers of health in ways that suggest it represents a tipping point in maintaining a separation of the socio-political from the individual.

In some group interviews, there were interesting snippets that hinted at more socially informed understandings of health determination, but these were rarely challenged head-on nor incorporated as part of the group repertoire. Instead, they existed as unsynthesised nuggets and the conversation would drift back uncontested to lifestyle-related issues. This is illustrated by one group, where one participant talked about the difficulty of individual change amidst poverty but the group narrative repeatedly returned to the importance of encouraging behaviour change.

Dissonance was also identified in the way that some social phenomena were discussed in terms of health determinants but not others. As we have highlighted, there were few sustained narratives about how policy decisions might create poor health or disparities in health, yet, many participants mentioned that people in poor circumstances and existing poor health were being made sicker by welfare reform:

“People who have maybe been on the sick, Employment Support Allowance for fifteen, eighteen years, and now they are being assessed and they are being basically put off the sick and they now have to claim Jobseekers Allowance and they are struggling. … The Jobcentre are putting so much pressure on them now that they are actually making people mentally ill.” (COP:I2)

It is possible that welfare reform in the UK, through media attention and popular dissent, has become somehow more visible as an emblem of unfair policy making to our participants than the wider machinery of economic and social policy-making. As a demonstration of disrupted narrative, it is again indicative of space for debate and utopian thinking in Levitas’ terms (Levitas, 2013).

## 5 Limitations

Before stating our conclusions, we highlight the main limitations of our study. We focus on three issues: the nature of our sample, the data collected and potential limitations of the narrative interview method.

First, the GPs that we spoke to were all, to varying degrees, engaged in the Deep-End network, a self-organised group working in the most socioeconomically deprived areas of Scotland. As a group, GPs at the Deep End produce reports campaigning for social justice and advocating the Scottish Government for resources to be directed towards their communities (General Practitioners at the Deep End, 2017; GPs at the Deep End, 2010). It is not clear how representative the views in this study are of GPs more widely, though arguably involvement in the Deep End group should be associated with greater structural competency rather than less (Babbel et al., 2017).

Second, as we set out earlier, we did not gather data on participant socio-demographics or biographic backgrounds – these, alongside more space to explore perspectives on class positionality, would have further enriched our analysis.

Third, we are conscious that narrative interviews *create* data. In understanding the possible contradiction in the narratives of those who simultaneously argued that politics create health and that social prescribing might reduce inequalities in health, it is important to remember that these interviews were conducted for evaluation purposes. It is conceivable that some narratives entailed a *performed* commitment to the programme and its continued funding. Performance is also at issue when it comes to group interviews where pre-existing personal and professional tensions, invisible to the researcher, may be enacted (Kitzinger and Barbour, 1999). It is also plausible that the disrupted discourses described are a feature of the research method – in discussion we rarely produce highly synthesised accounts of complex phenomena, particularly when moving between explanations at a population and an individual level.

Finally, we highlight that the data collected do not necessarily tell us how professionals interact with patients in consultations – potentially stigmatising discourse produced in a research interview may not be reproduced in actual professional-patient encounters – observational studies such as those conducted by Lutfey and Freese (2005) are required to determine the extent to which health protecting resources are strengthened or undermined in such micro-level encounters.

## 6 Conclusion

This paper started by setting out Scott-Samuel and Smith's (2015) proposition that interventions currently aimed at tackling HIs are flawed in ways that mean they *cannot* reduce the health gradient and that believing in them in these terms is ‘fantastical’. It examined the discourses of those engaged in delivering healthcare and/or a social prescribing programme in GP practices serving areas of multiple deprivation in Scotland, in relation to how such programmes might operate to address poor health and HIs. Our findings are congruent with the existing literature but provide significant new, empirically driven insights. Here we highlight five aspects where original contributions are made with important consequences for advancing the field in terms of research, policy and practice.

First, we offer a new analysis of why those who do not believe that health is a business of individual choice can find ways of supporting *fantasy paradigms* as embodied in social prescribing. We identify the categories: hopeful pessimist; supporters of mitigation at an individual level as worthwhile in its own right; and, those who acknowledge the social-determinants-of-health without connecting these to actual lives.

Second, our data (like those of a growing body of literature e.g. (Mackenzie et al., 2017a; Mackenzie et al., 2017b; Smith and Anderson, 2017; Roy, 2017) illustrate contested discourses on the operation of the social and political determinants of health. Recognition of the importance of macro-economic policy, for example, sits cheek-by-jowl with narratives of how changing lifestyle behaviours will reduce HIs. Our contribution to the literature is to identify *reasons for this disjuncture*. Some of these chime with those given by Scott-Samuel and Smith for researcher engagement with health improvement evaluations—because they are a source of funding—but we argue too that there is something about the pervasive weight of neoliberal rhetoric and practices, that actively creates a cleavage between the social/population perspective and the individual/clinical. This results for some in the *social-determinants-of health* being recognised in an amorphous (de-politicised way) then bracketed off from active understanding of *how the social and political actually determine the health of individuals and communities* served by a GP practice. Re-engaging communities of practice with what is meant by *determination* is, we argue, of key importance.

Our third conclusion relates to whether such interventions offer opportunities to better understand the actual social determinants of the embodied health of patients and, therefore, protect patients from stigmatising discourse (Babbel et al., 2017); our findings are though, disappointing in this respect. Although not the only discourse, most of the data pointed to lifestyle-oriented understandings of health problems and their solutions. Furthermore, for some GPs, burdened by workloads, social explanations were distractions to practicing within a medical model of health and CLPs viewed not as a means of encouraging structural understanding at practice level but of tidying up health presentations. Scott-Samuel and Smith argue that there is an unholy alliance between neo-liberalism as a set of processes, and traditional hierarchies of research methods that make complex social frameworks difficult to study; we suggest that a similar alliance with medical models of healthcare may serve the same function in relation to practice.

Our fourth conclusion is that, more positively, disjuncture offers a glimmer of hope for the existence of opportunities for democratic deliberations about health creation and its alternatives, as Scott-Samuel and Smith advocate. For at least some participants, these disrupted narratives suggest openness to engage in potentially fruitful discussions beyond the evaluation interview where performances of intervention support might have been enacted.

Our fifth conclusion cautions researchers, policymakers and practitioners about how the *fantasy paradigm* concept is understood and used. The intervention on which this paper is based grappled strategically with its role in tackling HIs and eventually stood back from its original aspirations that it would lead to reducing inequalities – instead, mitigation was viewed as the goal. Bracketing the question of whether social prescribing *works* in these terms (as we have indicated, whilst policy support is strong, the evidence base is meagre), there is an equally important question of how those delivering public services can be supported to do so in ways that are non-stigmatising for individual recipients. We argue that it is fundamentally important that public services are properly supported and legitimated as a means of strengthening safety nets for individuals especially when such safety nets are politically under sustained threat (Clifford, 2017; Hastings et al., 2015; Reich et al., 2016). This is particularly important where existing vulnerabilities have been compounded for already disadvantaged communities and groups in society (Asenova, 2015).

A more confident *de-coupling* of the policy aim of reducing HIs from that of delivery of structurally competent and equality-focused public services may offer the opportunity to strengthen the hand and validate the work of primary care and other services, to challenge problematic practices and, importantly, to limit obfuscating arguments about what is actually needed to reduce HIs.

## Author roles

This paper arises from the evaluation of the Link Worker Programme. MM was a part of the team responsible for designing the original study and managing data collection conducted by Nai Rui Chng. MM and KS took the lead in designing the analysis approach reported in the paper and this was implemented by MM, KS and GF. MM took the lead in drafting the paper with significant review from KS and editing from KS and GF.

## Footnotes

1 This paper uses the term health inequalities to refer to the systematic patterning of health outcomes by social group (referred to within the North American literature as health inequities). Unlike in Northern America, where disparities are patterned by race as well as class (Phelan and Link, 2015), in Scottish and UK contexts health inequalities refers predominantly (but not exclusively) to the significant inequalities experienced between different socio-economic groups (Babbel et al., 2017). Scotland has the worst such inequalities in Western Europe (Baruffati et al., 2019).
